# DAIR for periprosthetic joint infections—One week to save the joint?

**DOI:** 10.1186/s42836-024-00282-y

**Published:** 2024-12-05

**Authors:** Vatsal Gupta, Shafiq Shahban, Michael Petrie, Peter K. Kimani, Jakub Kozdryk, Bryan Riemer, Richard King, Richard Westerman, Pedro Foguet

**Affiliations:** 1 University Hospitals Coventry & Warwickshire NHS Trust, Coventry, CV2 2DX UK; 2 Warwick Medical School, University of Warwick, Coventry, CV2 2DX UK

**Keywords:** Infection, DAIR, Debridement, Antibiotics, Implant retention, Hip arthroplasty, Knee arthroplasty, Revision, Periprosthetic joint infection

## Abstract

**Background:**

Predicting the success of a Debridement, Antibiotics, and Implant Retention (DAIR) procedure for periprosthetic joint infection (PJI) for hip and knee joint arthroplasty remains a challenge. A failed DAIR might adversely affect the outcome of any future revision surgery for PJI. Hence, the ability to identify and optimize factors predictive of DAIR success would help target the procedure to the appropriate patient cohort and avoid unnecessary surgery for patients where a DAIR is unlikely to eradicate infection.

**Methods:**

A retrospective review of our prospective Bone Infection Group database was performed to identify all patients who underwent a DAIR of their primary or revision hip or knee arthroplasty. All patients had a confirmed PJI as per MSIS 2013 criteria and an outcome according to the MSIS working group outcome-reporting tool. DAIR surgery was then grouped into groups of “successful” or “unsuccessful” outcomes.

**Results:**

Sixty-four consecutive patients with an acute PJI underwent a DAIR procedure between 2009 and 2020, with 46 procedures performed for knees and 18 for hips. Treatment was successful in 69% (37 knees and 7 hips). The chance of a successful DAIR was significantly greater if performed at or within one week of symptom onset compared to greater than one-week duration (adjusted odds ratio (OR) 0.11; *P* = 0.027; 95% CI [0.02–0.78])). For DAIR performed at or within one week of symptom onset, the success rate was 93% for knees and 80% for hips. The chance of a successful DAIR however was not influenced by whether the surgeon was an arthroplasty or non-arthroplasty surgeon (OR 0.28; *P* = 0.13; 95% CI [0.05–1.48])). Isolated *Streptococcus* infection had a success rate of 100%. Next came Coagulase-negative *Staphylococci* (71%) and Methicillin-susceptible *Staphylococcus Aureus* (65%). Polymicrobial infection had the worst outcome, with a success rate of 40%.

**Conclusion:**

In our experience, DAIR surgery performed within one week of symptom onset significantly increased the chance of successful infection eradication. Collaborative work is required to ensure arthroplasty patients can access prompt appropriate surgical decision-making as soon as concerns arise, remove barriers to early assessment and minimise delays to surgery.

## Background

Prosthetic joint infection (PJI) is a devastating complication of primary joint arthroplasty with significant life-changing consequences and impaired quality of life for the patient [1].

PJI is the most common indication for revision of total knee arthroplasty (TKA) and the third most common for revision of total hip arthroplasty (THA) [2]. With an ever-increasing global demand for arthroplasty surgery, the burden of PJI is only set to rise. Projections from the USA for total joint arthroplasty (TJA) forecast a rise of 129–182% by 2030 and a further rise of 284–401% by 2040 (Singh) compared to 2014 [3]. With similar projected increases in demand worldwide [4], evidence-based and judicious management of PJI will be crucial.

The choice of surgical management of PJI remains complicated and is subject to many factors, requiring a multi-disciplinary approach [5]. The two surgical philosophies in managing PJI are either debridement and implant retention (DAIR) or the exchange of all components in either one or two stages [6]. A DAIR procedure is widely accepted as the preferable choice compared to component exchange when managing an acute PJI with well-fixed components. Acute infection is generally considered within four weeks of the index procedure. DAIR procedures carry the advantages of being a single procedure with a shorter period of hospitalization, lowered associated morbidity, and less cost [7]. It has, however, been suggested that the results of a component exchange revision may potentially be compromised in patients who have previously undergone a failed DAIR procedure [8]. It is, therefore, imperative to identify predictors of treatment success to ensure the appropriate patients are selected for DAIR procedures and controllable factors are optimized, thus preventing unnecessary and potentially compromising surgery in patients in whom a DAIR is unlikely to eradicate infection.

Different surgical and patient factors have been reported in literature to affect the success rates of DAIR procedures. These have included factors such as time between symptom onset and surgery, type of infecting organism, length of antibiotic use, immunocompromise, presence of a sinus, etc. [9, 10]. However, evidence has remained inconsistent, and a consensus has yet to be reached.

Success rates for DAIR performed for PJI of the hip were previously reported to be 70% and 63% for DAIR performed for PJI of the knee. Pooled overall success rate for DAIR for hip and knee PJI has been reported to be 67% [11]. A few scoring systems have also been proposed to predict failure after a DAIR procedure, such as the KLIC-score [12] for early acute PJI and the CRIME-80 score [13] for late acute PJI, however, the validity of both scores remains contentious.

This study aimed to explore and identify factors predictive of DAIR success which would then help target the procedure to the appropriate patient cohort and avoid unnecessary surgery for patients in whom a DAIR is unlikely to eradicate infection.

## Methods

For over ten years, the Bone Infection Group Coventry and Warwickshire (BIGCOW), as a multidisciplinary team, has managed the treatment of PJIs at a tertiary referral center. Since its inception, the database has collated patients with PJI, noting outcomes, microorganisms, sensitivities, surgical management, and antibiotic regimens. A retrospective review of our tertiary referral center’s prospectively-collated bone infection group (Bone Infection Group Coventry and Warwickshire [BIGCOW]) database was performed. All patients who underwent a DAIR procedure for confirmed acute PJI of a primary or revision hip or knee arthroplasty between 2009 to 2020 were included. Any patients undergoing a DAIR where, as a result of incomplete datasets or incorrect coding, a diagnosis of PJI could not be confirmed, were excluded.

All patients with confirmed infection by the Musculoskeletal Infection Society (MSIS) definition from 2013 [14] (Table 1) were included. For patients adjudged to not have an infection on the MSIS 2013, the results were also checked against the European Bone and Joint Infection Society (EBJIS) criteria 2021 [15] (Table 2). If they were considered infected by these criteria, they were also included in the study.

**Table 1:** Musculoskeletal Infection Society (MSIS) 2013 Criteria [14]

Musculoskeletal Infection Society (MSIS) criteria	
Periprosthetic joint infection is present when one major criterion is present or three out of five minor criteria exist	
Major Criteria	• Two positive periprosthetic cultures with phenotypically identical organisms • A sinus tract communicating with the joint
Minor criteria	• Elevated CRP and ESR • Elevated synovial fluid WBC count or + + change on leukocyte esterase strip • Elevated synovial fluid PMN% • Positive histological analysis of periprosthetic tissue • A single positive culture

**Table 2:** European Bone and Joint Infection Society (EBJIS) 2021 “Infection Likely” Criteria [15]

**European Bone and Joint Infection Society (EBJIS) 2021 Infection Likely Criteria**		
**Periprosthetic joint infection is present when there are two positive findings** **A + B OR A + C**		
A Clinical	Clinical Features	• Early radiographic loosening • Wound healing problems • Recurrent fever/ Bacteraemia • Purulence around prosthesis
A Clinical	CRP	• CRP > 10 mg/L
B Laboratory	Synovial fluid	• Leukocyte count > 1500 • PMN > 65%
B Laboratory	Microbiology	• Single Positive Culture (aspiration or intra-operative) • > 1 CFU/mL any organism on sonication
B Laboratory	Histology	• Presence of ≥ 5 neutrophils in a single HPF
C Radiology	Nuclear Imaging	• Positive white cell labelled scintigraphy

Patient demographics, clinical data, medical and surgical management, and laboratory results were extracted from local electronic hospital patient records. Surgical outcomes were recorded according to the MSIS working group outcome-reporting tool; and then grouped into either “successful” (infection control with no continued antibiotic treatment, further aseptic revision, or death after 1 year) or “unsuccessful” (suppressive antibiotics, further revision for infection, or death within 1 year).

Timing of surgery was calculated as the interval between initial symptom onset and performance of the DAIR procedure. Grade of the surgeon performing the DAIR procedure was categorized into three groups: Revision Arthroplasty, Arthroplasty, or Non-arthroplasty, depending upon the individual surgeon’s subspecialist experience.

Once the decision to perform a DAIR was made, the surgery was performed on the next available trauma or elective operating list. The DAIR was performed in the supine position for knees and lateral decubitus position for hips. The DAIR consisted of an initial exposure of the joint utilizing the previous skin incision followed by an aggressive, thorough, and systematic synovectomy and debridement of all possibly infected tissues down to the prosthesis. Implants were checked for their stability, which was followed by an exchange of all modular components. During the procedure, multiple tissue samples (aiming for 5 samples with a clean set of instruments for each sample) for microbiological analysis were collected and sent expeditiously from theatre as soon as all samples were taken. Absorbable calcium sulphate beads with 1 g vancomycin and 240 mg gentamicin per 10 cc mix (Stimulan, Bicomposites, Keele Science Park, Staffordshire, England, ST5 5NL) were placed in the joint cavity as an additional way of delivering local antibiotics and closure was meticulously performed. All postoperative microbiological decisions were made in our formal bone infection group (BIGCOW) MDT meeting, involving revision arthroplasty surgeons, microbiologists, infectious disease specialists, and pharmacists.

Our antibiotic therapy protocol following a DAIR consisted of initial dual broad-spectrum intravenous antibiotics with vancomycin (calculated using a vancomycin dosing calculator) and meropenem (1 g three times a day) with subsequent tailoring once culture results are known, thereby ensuring the antibiotic regime was biofilm-penetrative with all regimens discussed at our BIGCOW MDT with microbiological and pharmaceutical input. The typical duration of total antibiotic therapy would last 6–12 weeks, including initial inpatient and outpatient intravenous antibiotics, aiming for subsequent conversion to oral therapy based on clinical and biochemical improvement. For patients with negative cultures and no sensitivities, broad spectrum combination with vancomycin and meropenem was used postoperatively whilst applying our previously published diagnostic algorithm to identify an organism [16].

### Statistical analysis

Mean (standard deviation), median (lower quartile–upper quartile), minimum, and maximum were calculated to summarize the age of the cohort. Other patient characteristics were categorical, and they were summarized in terms of count and percentage of patients in each category. DAIR success rate in each category was also reported. Univariable (unadjusted analysis) and multivariable (adjusted analysis) logistic regression models were fitted to determine predictors for DAIR success. Odds ratios were computed, with a value greater than 1 indicating greater odds of a successful DAIR procedure compared to reference category while odds ratio less than 1 indicating lesser odds of a successful DAIR compared to the reference category. A *P*-value of < 0.05 indicated a significant predictor for DAIR success.

## Results

Sixty-four consecutive patient records that met our inclusion criteria were identified on interrogation of the BIGCOW database. All patients had received a DAIR procedure for confirmed PJI following a primary or revision hip or knee Joint Arthroplasty.

Forty-six (72%) DAIR procedures were performed for primary or revision arthroplasty of the knee. Of these, 39 were performed for primary knee arthroplasty and seven were performed for revision knee arthroplasty.

18 (28%) DAIR procedures were performed for primary or revision arthroplasty of the hip. Of these, 16 were performed for primary hip arthroplasty and two were performed for revision hip arthroplasty procedures.

In our series, 57 patients (89%) were confirmed as infected according to the Musculoskeletal Infection Society (MSIS) (2013) criteria (Table 1). We further increased the number of cases in our series by 7 additional patients who, according to EBJIS 2021 diagnostic criteria were “infection likely” (Table 2). The mean age of the patients was 68 years (range 37–92) and 39 (61%) patients were male (Table 3). Patients were subsequently categorized based on their outcomes using the MSIS working group outcome-reporting tool (Table 4) [17]. Success of DAIR surgery evaluated as per the MSIS reporting outcome tool is illustrated in Table 5 [17].

**Table 3:** Summary of results

Characteristic	*n* (%)	Success (%)	Odds ratio (95 confidence interval), *P*-value	
			**Unadjusted**	**Adjusted**
**Age**				
Minimum–Maximum	37.3–92.4	-	1.00 (0.96–1.05), 0.900	-
Median (LQ–UQ)	70 (59–78)			
Mean (Standard deviation)	68.7 (12.6)			
**Gender**				
Male	39 (60.9)	28 (71.8)		-
Female	25 (39.1)	16 (64.0)	0.70 (0.24–2.04), 0.512	
**Surgeon**			0.339	0.303
Arthroplasty	24 (37.5)	19 (79.2)	Reference category	Reference category
Revision	26 (40.6)	17 (65.4)	0.50 (0.14–1.78), 0.282	0.46 (0.10–2.15), 0.326
Non-arthroplasty	14 (21.9)	8 (60.0)	0.35 (0.08–1.49), 0.156	0.28 (0.05–1.48), 0.134
**Organism prior to DAIR**				
No	41 (64.1)	28 (68.3)		-
Yes	23 (35.9)	16 (69.6)	1.06 (0.35–3.20), 0.916	
**Symptom duration**				
≤ 1 Week	20 (31.7)	18 (90.0)	Reference category	
> 1 Week	43 (68.3)	25 (58.1)	0.15 (0.03–0.75), 0.021	0.11 (0.02–0.78), 0.027
**KLIC score**			0.057	0.066
0–2	21 (32.8)	13 (61.9)	Reference category	Reference category
2.5–3.5	11 (17.2)	8 (72.7)	1.64 (0.33–8.07), 0.542	1.48 (0.25–8.57), 0.664
4–5	20 (31.3)	18 (90.0)	5.54 (1.01–30.5), 0.049	3.76 (0.58–24.3), 0.164
5.5–8.0	12 (18.8)	5 (41.7)	0.44 (0.10–1.87), 0.265	0.21 (0.03–1.54), 0.126

**Table 4:** Patients categorised according to MSIS working group outcome-reporting tool [17]

**Tier 1**	Infection control with no continued antibiotic treatment	35
**Tier 2**	Infection control with suppressive antibiotic treatment	3
**Tier 3**	Need for reoperation and/or revision and/or spacer	
	• Aseptic revision	1
	• Septic revision	13
**Tier 4**	Death (assigned to subgroups of A or B)	
**A**	• Death < 1 year from surgery	4
**B**	• Death > 1 year from surgery	8
Total		64

**Table 5:** Outcomes of DAIR surgery according to MSIS working group outcome-reporting tool [17]

**Successful DAIR**	
• Infection control with no continued antibiotic treatment	35
• Further aseptic revision	1
• Death > 1 year from DAIR	8
Total	44
**Unsuccessful DAIR**	
• Further septic revision	13
• Infection control with suppressive antibiotic treatment	3
• Death < 1 year from DAIR	4
Total	20

Of the 46 DAIR procedures performed for PJI of primary or revision arthroplasty of the knee, 37 were successful, giving a success rate of 80%. Of these 37, thirty-two were successful DAIR performed for primary knee arthroplasty and five were successful DAIR performed for revision procedures.

Of the 18 DAIR procedures performed for PJI of primary or revision arthroplasty of the hip, seven were successful, yielding a success rate of 39%. Of the 18, all the DAIR that were successful, were performed for primary hip arthroplasty (seven) whilst the two DAIR performed for revisions procedures of the hip were unsuccessful.

Overall pooled success rate for DAIR performed for both hip and knee primary and revision arthroplasty was 69% (44 patients) and DAIR was unsuccessful in 31% (20 patients). The overall success rate for DAIR performed for primary arthroplasty procedures was 70% (39/44). Of the 9 patients undergoing DAIR for revision arthroplasty, the overall success rate was 56% (5/9).

The chances of a successful DAIR were significantly greater if performed at or within 1 week of symptom onset compared to greater than 1-week duration (90% vs. 58.1%; adjusted odds ratio (OR) 0.11; *P* = 0.027; 95% CI [0.02–0.78]). Of the DAIR procedures performed within 1 week duration, 15 were performed for PJI of the knee and five were for PJI of the hip. The success rate of DAIR for PJI of the knee performed at or within 1 week of symptom onset was 93% (14/15) and those for the hip was 80% (4/5). Patients who underwent their DAIR within one week of symptom onset had a success rate of 88.2% (15/17), which dropped to 62.2% (23/37) between one and four weeks and to 56% (5/9) when performed more than four weeks following onset.

Arthroplasty surgeons achieved a success rate of 80% (16/20) for DAIR procedures of the knee and 75% (3/4) for DAIR procedures of the hip. Revision arthroplasty specialists attained a success rate of 70% for DAIR procedures of the knee and 50% (3/6) for DAIR procedures of the hip. Non-arthroplasty surgeons had a success rate of 77% (7/9) for DAIR procedures of the knee and 20% (1/4) for DAIR procedures of the hip. The pooled success rates of DAIR procedures for both hip and knee were found to be 79.2% (19/24) when performed by arthroplasty surgeons, 65% (17/26) when undertaken by a revision arthroplasty specialist, and 60% (8/14) for non-arthroplasty surgeons. Revision arthroplasty surgeons performed a greater proportion of DAIR procedures for infected revision arthroplasty and arthroplasty for fracture of the femoral neck (29%). The chances of a successful DAIR were not influenced by whether the surgeon was an arthroplasty or non-arthroplasty surgeon (adjusted OR 0.28; *P* = 0.13 95% CI [0.05–1.48]).

Streptococcus infection was identified in ten cases in our cohort (involving 8 knees and 3 hips), with a DAIR success rate of 100%. Seven cases were identified to have isolated coagulase-negative Staphylococcus infections, all within the knee joint. A DAIR success rate of 71% (5/7) was achieved for this organism. Isolated methicillin-sensitive Staphylococcus Aureus was found in 17 cases (6 hips, 11 knees). DAIR was successful in 33% of hips and 82% of knees with regards to this organism, with the overall success rate being 65% (11/17). Gram-negative infection was found to have a success rate of 60% (3/5), with polymicrobial infection demonstrating the worst outcome, with a success rate of 40% (6/15). Culture-negative PJI was seen in 12.5% (8/64) of cases, all within the knee, with 88% (7/8) of these patients proceeding to a successful outcome of their DAIR procedure.

An inverse correlation was identified between the KLIC score and the outcome. Patients with a higher KLIC score (and thus theoretically predicting a higher risk of failure) of between 4–5 (*n* = 20) had a success rate of 90% (18/20) compared to just 62% (13/21) for those with the lowest score (0–2) (adjusted OR 3.76; *P* = 0.164 95% CI [0.58–24.3]).

## Discussion

The successful management of PJI remains complex and difficult to predict [1].

The exact time frames and associated success rates of DAIR procedures have continued to invite debate [18–20]. It is widely accepted, however, that a DAIR is recommended for the treatment of acute PJIs. Contention has surrounded the definition of an “acute” infection, [21] however, the greater the time interval from surgery the longer a biofilm could opportunistically develop, thereby decreasing the chances of success [22]. Acute postoperative infection is generally considered within four weeks of the index procedure, whereas late acute infection tends to be an acute infection after a previous successful arthroplasty, invariably due to hematogenous spread.

Hartman et al. in 1991, in a cohort of 33 patients with infected TKA, reported a significant improvement in success rates for DAIR procedures performed within four weeks duration [18]. Similarly, Qu et al., in their pooled analysis of 1266 cases of prosthetic knee infections, reported no significant difference in success rates between seven days and three weeks for DAIR procedures but noted a sharp decrease in success rates beyond the latter [19]. Sendi et al. also reported comparable findings in their analysis of 34 cases of PJI after THA attaining a 91% success rate where the duration of symptoms did not exceed three weeks [20].

This prospectively collected series found that when DAIR surgery was performed at or within one week of symptom onset, there was a significant increase in successful outcomes (adjusted OR 0.11; *P* = 0.027; 95% CI [0.02-0.78]). In our cohort, 15 DAIR procedures were performed for PJI of the knee and five were performed for PJI of the hip at or within 1 week duration of symptom onset. The success rate of DAIR for PJI of the knee in these cases was 93% (14/15) and those performed for the hip was 80% (4/5). This finding is corroborated by Tsang et al., who, in their meta-analysis of infected THA in 2017, found the success rate to be significantly greater when performed within seven days of symptom onset [23].

The experience of the surgeon performing a DAIR procedure has been debated as an individual factor predictive of its success. Iza et al. reported a higher success rate of DAIR procedures (77%) when all procedures were performed by arthroplasty surgeons [24]. Conversely, Young et al. looked at surgeon involvement in a DAIR procedure as an isolated factor and reported that the presence of an arthroplasty surgeon in theatre for a DAIR did not reduce the risk of failure [25]. Similarly, we also reported that the chances of a successful DAIR were not influenced by whether the DAIR procedure was performed by an arthroplasty or non-arthroplasty surgeon (adjusted OR 0.28; *P* = 0.13 95% CI [0.05–1.48]). However, it must be noted that the success rate of DAIR procedures was higher for arthroplasty and revision arthroplasty surgeons compared to non-arthroplasty surgeons (72% vs. 57%), and although it did not reach statistical significance, this could be ascribed to the small sample size. Future larger studies are then warranted to assess this with more precision.

Superior infection control rates for Streptococcal infection, as compared to other microbes, are well documented in literature [26, 27]. We too reported a 100% success rate for isolated Streptococcus infection; followed by coagulase-negative Staphylococci (71%) and methicillin-susceptible *Staphylococcus Aureus* (65%). Polymicrobial infection had the worst outcome with a success rate of 40%. The latter is supported by Lora-Tamayo et al., who highlighted that patients with polymicrobial PJI had worse outcomes and were less likely to have a successful DAIR, necessitating the need for formal revision surgery [28].

In this series, 12.5% (8/64) received a DAIR for culture-negative PJI, all for arthroplasty of the knee, with 88% (7/8) of these patients proceeding to a successful outcome of their DAIR procedure. No microorganisms were identified on the culture of either their aspirate (performed in 6/8) or tissue samples sent at the time of DAIR. We hypothesize that preoperative administration of antibiotics in several of these patients may have affected subsequent tissue culture results. This hypothesis is supported by the work of Malekzadeh et al. in 2010, who reported that 64% of their group (135 culture-negative patients) had received antimicrobial therapy within the 3 months prior to specimen retrieval [29].

Our study reported a success rate of 80% for DAIR procedures performed for primary and revision knee arthroplasty and a 39% success rate for primary and revision hip arthroplasty. This differed from the findings of Gerritsen et al. Their recent systematic review, including 65 studies and encompassing 6,630 patients, reported a success rate of 70% for hips and a success rate of 63% for knees (with some overlap in confidence intervals) [30]. However, it is important to consider limitations in this context. Our study had a much higher proportion of DAIR procedures for knee arthroplasty (72%) against DAIR for hips (28%). Additionally, unsuccessful hip DAIR procedures were more likely to have been performed for trauma compared to unsuccessful knee DAIR procedures (none of which were performed for trauma). This lack of homogeneity between groups thus made it difficult to draw any meaningful inferences about the success rate variations. Further research with more balanced patient groups is needed in this regard.

The KLIC score has previously undergone external validation and was found to demonstrate inconsistent correlation between a score and prognosis following a DAIR [31]. The same group did, however, highlight that patients with a score ≥ 7 were at high risk of failure. Sabater-Martos et al. went one step further and showed that the KLIC score had no predictive value [32]. Further evidencing an inconsistent correlation with the KLIC score, we also found no correlation between the KLIC score and treatment success. Patients with a high KLIC score of between 4–5 (*n* = 21) had a success rate of 90% compared to just 62% (for those with the lowest score (0–2) (adjusted OR 3.76; *P* = 0.164 95% CI [0.58–24.3]). Only one patient had a KLIC score within the highest tier (KLIC score = 8) and yet their treatment was still successful. The rate of liver failure within our population was very low (1 patient), so, although this patient’s DAIR was unsuccessful, this component of the scoring system added little value. Most of the patients in our series had a cemented prosthesis at index surgery (84%). Although this proportion was greater in the unsuccessful group (90% vs. 82%), this component of the scoring system was a poor differentiator. Pre-existing kidney failure was more prevalent in the unsuccessful group (30%) compared to the successful group (14%) suggesting an association with a less successful outcome. Boyer et al. reported that pre-existing kidney and/or liver failure were suggestive of a poor outcome following a DAIR [33].

There are limitations to this single-centre study. Due to the relatively small sample size, there existed some degree of uncertainty in the results, as reflected by the wide 95% confidence intervals.

Between 2009 and 2020, we performed many more DAIR procedures than the ones included in our series that unfortunately did not satisfy the inclusion criteria and this is a common problem of retrospective studies. Larger multi-center studies in the future should address this issue and hopefully confirm our findings. Another possible limitation of the study being carried out in a tertiary referral center is the potential for missed early data for patients treated in neighbouring hospitals prior to onward referral. However, with the majority of our referring hospitals utilizing linked electronic systems, this is likely negligible.

A key strength of this study was its prospectively collated database from the BIGCOW group. Consistent MDT decision-making is ensured, helping enrich data and reduce variability, which may be a limitation of multi-center studies. Another advantage of this series from a single centre is that our philosophy of aggressive debridement techniques and bone infection group management reduces the significant variance in DAIR management. As a result, we are better able to focus on the variable of time interval until surgery.

Future collaborative work is required between clinicians in primary and secondary care to ensure arthroplasty patients can access prompt appropriate surgical decision-making, as soon as concerns arise, to remove barriers to their early clinical assessment and minimize delays to surgery.

## Conclusion

PJI remains a significant and catastrophic postoperative complication following primary hip and knee arthroplasty. Our findings suggest that the chances of a successful DAIR are significantly greater when performed within one week of symptom onset. Larger multi-center studies are required to confirm our findings.

## Data Availability

The datasets used and/or analyzed during the current study are available from the corresponding author on reasonable request.

## Abbreviations

DAIR: Debridement, Antibiotics and Implant retention
PJI: Periprosthetic Joint Infection
THA: Total Hip Arthroplasty
TKA: Total Knee Arthroplasty
NJR: National Joint Registry
